# A Quantified Coalgebraic van Benthem Theorem

**DOI:** 10.1007/978-3-030-71995-1_28

**Published:** 2021-03-23

**Authors:** Paul Wild, Lutz Schröder

**Affiliations:** 8 University of Oxford, Oxford, UK; 9 Sorbonne Université - LIP6, Paris, France; Friedrich-Alexander-Universität Erlangen-Nürnberg, Erlangen, Germany

**Keywords:** Modal logic, Quantale, Fuzzy logic, Coalgebra, Behavioural distance, Modal characterization.

## Abstract

The classical van Benthem theorem characterizes modal logic as the bisimulation-invariant fragment of first-order logic; put differently, modal logic is as expressive as full first-order logic on bisimulation-invariant properties. This result has recently been extended to two flavours of quantitative modal logic, viz. fuzzy modal logic and probabilistic modal logic. In both cases, the quantitative van Benthem theorem states that every formula in the respective quantitative variant of first-order logic that is bisimulation-invariant, in the sense of being nonexpansive w.r.t. behavioural distance, can be approximated by quantitative modal formulae of bounded rank. In the present paper, we unify and generalize these results in three directions: We lift them to full coalgebraic generality, thus covering a wide range of system types including, besides fuzzy and probabilistic transition systems as in the existing examples, e.g. also metric transition systems; and we generalize from real-valued to quantale-valued behavioural distances, e.g. nondeterministic behavioural distances on metric transition systems; and we remove the symmetry assumption on behavioural distances, thus covering also quantitative notions of simulation.

### 1 Introduction

Modal logic takes part of its popularity from the fact that it specifies transition systems at what for many purposes may be regarded as the right level of granularity; that is, it is invariant under the standard process-theoretic notion of *bisimulation* in the sense that bisimilar states satisfy the same modal formulae. There are two quite different well-known converses to this elementary property, which both witness the *expressiveness* of modal logic: By the *Hennessy-Milner theorem* [29], states in finitely branching systems that satisfy the same modal formulae are bisimilar, and by the *van Benthem theorem*, every first-order definable bisimulation-invariant property is expressible by a modal formula. Since modal logic embeds into first-order logic, the latter result may be phrased as saying that modal logic is the bisimulation-invariant fragment of first-order logic.

⋆ Work of both authors forms part of the DFG project *Probabilistic description logics as a fragment of probabilistic first-order logic* (SCHR 1118/6-2)

In the two-valued setting, there has been increased recent interest in variants and generalizations of this result (e.g. [54,14,52,22,55,1])

For quantitative systems, it has long been realized (e.g. [26,15,10]) that quantitative notions of process equivalence, generally referred to as *behavioural metrics* (although they are in general only *pseudo*metrics, as distinct but equivalent states have distance zero), are often more appropriate than two-valued bisimilarity. In particular, while two-valued notions of process equivalence just flag small deviations between systems as inequivalence, behavioural metrics can provide more fine-grained information on the degree of similarity of systems. Behavioural metrics are correspondingly used, e.g., in verification [25], differential privacy [13], and conformance testing of hybrid systems [36].

In the same way that two-valued modal logic constitutes a natural specification language for two-valued transition systems, quantitative systems correlate to quantitative modal logics. In this context, bisimulation invariance is read as *nonexpansiveness* w.r.t. behavioural distance, i.e. two states differ on a modal formula at most by their behavioural distance; we refer to this property as *behavioural nonexpansiveness*. Notably, van Breugel and Worrell [10] prove a Hennessy-Milner type theorem for a quantitative probabilistic modal logic: They show that on compact state spaces, the formulae of the logic lie dense in the space of behaviourally nonexpansive state properties, which implies that behavioural distance and logical distance coincide.

In the present paper, we are mainly interested in the other converse to behavioural nonexpansiveness, i.e. in *quantitative van Benthem theorems*. In previous work with Pattinson and König, we have established such theorems for quantitative modal logics of fuzzy [57] and probabilistic [58] transition systems. In the quantitative setting, these theorems take the form of approximability properties, and state that every behaviourally nonexpansive quantitative first-order property is approximable by quantitative modal formulae *of bounded rank*. The latter qualification is in fact the key content of the respective theorems – without it, approximability is closer in flavour to Hennessy-Milner-type theorems, which apply to arbitrary rather than just first-order definable properties (although one should note additionally that our van Benthem theorems do not assume compactness of the state space).

Our present contribution is to unify and generalize these results in three directions: First, we allow for full *coalgebraic generality*, i.e. we cover system types subsumed under the paradigm of *universal coalgebra* [49]. Besides the fuzzy and probabilistic systems featuring in the previous concrete instances of our result, this includes a wide range of weighted, game-based, and preferential systems; for illustration, we concentrate on the (comparatively simple) case of *metric transition systems* [3,20] in the presentation. Second, we generalize from real-valued to *quantale-valued* metrics (e.g. [24,33]). Using the unit interval quantale, we recover our previous results on real-valued logics as special cases. Beyond this, quantales in particular provide support for what may be termed *metrics with effects*; we illustrate this on a notion of *convex-nondeterministic behavioural distance* on metric transition systems, where the behavioural distance gives an interval of possible real-valued distances. Lastly, we remove the assumption that distances need to be symmetric, so that we cover also notions of quantitative simulation. At this level of generality, we prove both a Hennessy-Milner type theorem stating coincidence of logical and behavioural distance, effectively generalizing the existing coalgebraic quantitative Hennessy-Milner theorem [37] to quantale-valued distances; and, as our main result, a quantitative van Benthem theorem stating that all behaviourally non-expansive first-order properties can be modally approximated in bounded rank.

*Related Work* There is a substantial body of work on two-valued modal characterization theorems, e.g. for logics with frame conditions [14], coalgebraic modal logics [52], fragments of XPath [12,22,1], neighbourhood logic [28], modal logic with team semantics [38], modal $\mu$-calculi (within monadic second order logics) [35,19], PDL (within weak chain logic) [11], modal first-order logics [6,54], and two-dimensional modal logics with an $S5$-modality [55]. We are not aware of quantitative modal characterization theorems other than the mentioned ones for fuzzy and probabilistic modal logics [57,58]. Prior to the quantitative Hennessy-Milner theorems mentioned above [10,37], Hennessy-Milner theorems have been established for *two-valued* logics and two-valued bisimilarity over quantitative systems, e.g. on probabilistic transition systems [39,16,17]. There is work on Hennessy-Milner theorems for certain Heyting-valued modal logics [21,18]; since Heyting algebras are quantales but often fail to meet a continuity assumption needed in our generic Hennessy-Milner theorem, we do not claim to subsume these results.

### 2 Preliminaries

We briefly recall basic definitions and examples on quantales and universal coalgebra, and fix some data needed throughout the paper. We need some elementary category theory, see, e.g., [2].

**Quantales** are order-algebraic structures that serve as objects of truth values in suitable multi-valued logics, and also support a useful notion of generalized (pseudo-)metric space (e.g. [24,33,32]). Our arguments will rely on a certain amount of epsilontics, and hence require more specifically the use of *value quantales* [24].

We recall some basic order and lattice theory. A *complete lattice* is a partially ordered set $(V, \leq)$ having all suprema $\bigvee A$ for $A \subseteq V$, equivalently all infima $\bigwedge A$. We denote binary meets and joins by $\wedge$ and $\vee$, respectively. Given $x, y \in V$, we say that $x$ is *well above* $y$, and write $x \gg y$, if whenever $y \geq \bigwedge A$ for some $A \subseteq V$, then $x \geq a$ for some $a \in A$. A complete lattice $(V, \leq)$ is *completely distributive* if all joins in $V$ distribute over all meets, equivalently all meets distribute over all joins [46]. Another equivalent characterization is that $(V, \leq)$ is completely distributive iff

$$y = \bigwedge\{x \in V \mid x \gg y\}$$

for every $y \in V$ [47].

In the definition of value quantale, we follow Flagg [24] in dualizing the usual continuity condition for quantales in order to avoid having to reverse the order when moving between the general development and basic examples such as the unit interval; deviating from his terminology, we emphasize this by the prefix 'co-':

**Definition 2.1 ((Value) co-quantales).** A *(commutative) co-quantale* $\mathcal{V}$ is a complete lattice $(V, \leq)$ equipped with a commutative monoid structure $(\mathsf{0}, \oplus)$ that is *meet-continuous*:

$$a \oplus \bigwedge_{i \in I} b_i = \bigwedge_{i \in I} (a \oplus b_i).$$

A co-quantale $\mathcal{V}$ is a *value co-quantale* [24] if $\mathsf{0}$ is the bottom element of $\mathcal{V}$ and moreover $(V, \leq)$ is a *value distributive lattice*, i.e. a completely distributive complete lattice such that $|V| > 1$ and for all $x, y \in V$, $x, y \gg \mathsf{0}$ implies $x \wedge y \gg \mathsf{0}$. Correspondingly, we denote the greatest element of $\mathcal{V}$ by $\mathsf{1}$.

(Dually, in a *quantale* the operation $\oplus$ is required to be *join-continuous.*) By meet-continuity, we obtain a further binary operator $\ominus$ on a co-quantale $\mathcal{V}$ by adjunction, defined by

$$a \ominus b \leq v \quad \text{iff} \quad a \leq b \oplus v$$

(equivalently, $a \ominus b = \bigwedge \{v \mid a \leq b \oplus v\}$). The operator $\ominus$ is sometimes called the *internal hom* of $\mathcal{V}$ [7]. Moreover, in a value co-quantale, we have that for each $\varepsilon \gg \mathsf{0}$, there exists $\delta \gg \mathsf{0}$ such that $2 \cdot \delta := \delta \oplus \delta \leq \varepsilon$ [24, Theorem 2.9]. This allows for proofs where an error bound $\varepsilon \gg \mathsf{0}$ needs to be split up into multiple smaller parts.

A simple example of a value co-quantale is the unit interval $[0, 1]$ with the usual ordering, with truncated addition $a \oplus b = \min(a + b, 1)$ as the monoid structure. Correspondingly, the $\ominus$ operation is truncated subtraction $a \ominus b = \max(a - b, 0)$. We have $a \gg b$ iff $a > b$. We will give further examples in Section 3.

**Universal Coalgebra** serves as a unified framework for many types of state-based systems [49], such as nondeterministic, probabilistic, alternating, game-based, or weighted systems. It is based on encapsulating the system type as a *functor* $T$, for our purposes on the category $\mathsf{Set}$ of sets and functions; such a $T$ assigns to each set $X$ a set $TX$, thought of as a type of structured collections over $X$, and to each map $f \colon X \to Y$ a map $Tf \colon TX \to TY$, respecting identities and composition. A *$T$-coalgebra* $(A, \alpha)$ consists of a set $A$ of *states* and a *transition map* $\alpha \colon A \to TA$, thought of as assigning to each state a structured collection of successors. Taking $T$ to be the *covariant powerset functor* $\mathcal{P}$, which assigns to each set $X$ its powerset $\mathcal{P}X$, we obtain relational transition systems as $T$-coalgebras. As a further example, the *(discrete) subdistribution functor* $\mathcal{S}$ assigns to each set $X$ the set $\mathcal{S}X$ of discrete probability subdistributions $\mu$ on $X$ (i.e. $\mu(X_0) = \mu(X) \leq 1$ for some countable subset $X_0 \subseteq X$), and to each map $f \colon X \to Y$ the image measure function (i.e. $\mathcal{S}f(\mu)(B) = \mu(f^{-1}[B])$ for $B \subseteq Y$).

$\mathcal{S}$-coalgebras are probabilistic transition systems (or Markov chains) with possible deadlock: They assign to each state a subdistribution over possible successor states, with the gap of the total probability to 1 interpreted as the probability of deadlock. Additional instances are seen in Example 4.4. For the remainder of the paper, we *fix a set functor $T$ and require that $T\emptyset$ is nonempty* (hence our use of subdistributions instead of distributions in the examples). Moreover, we require w.l.o.g. that $T$ is *standard*, i.e. preserves subset inclusions [5].

### 3 Quantale-Valued Distances and Lax Extensions

A *$\mathcal{V}$-valued relation* between sets $A$ and $B$ is a map $R\colon A \times B \to V$, which we also denote by $R\colon A \nrightarrow B$. For fixed $A$ and $B$, we order the $\mathcal{V}$-valued relations between $A$ and $B$ pointwise: $R_1 \leq R_2 \iff \forall a \in A, b \in B.\ R_1(a,b) \leq R_2(a,b)$. We compose relations $R\colon A \nrightarrow B$ and $S\colon B \nrightarrow C$ using the monoid operation on $\mathcal{V}$:

$$(R;S)(a,c) = \bigwedge\{R(a,b) \oplus S(b,c) \mid b \in B\}.$$

Given a function $f\colon A \to B$ and $\varepsilon \in V$, the *$\varepsilon$-graph* $\mathrm{Gr}_{\varepsilon,f}$ is the relation

$$\mathrm{Gr}_{\varepsilon,f}(a,b) = \begin{cases} \varepsilon, & \text{if } f(a) = b; \\ 1, & \text{otherwise.} \end{cases}$$

We also write $\mathrm{Gr}_f = \mathrm{Gr}_{\mathsf{0},f}$ and, in case of the identity function, $\Delta_{\varepsilon,X} = \mathrm{Gr}_{\varepsilon,\mathsf{id}_X}$ and $\Delta_X = \Delta_{\mathsf{0},X}$.

**Definition 3.1 ($\mathcal{V}$-continuity space).** Let $X$ be a set and let $d\colon X \nrightarrow X$. The pair $(X,d)$ is a *$\mathcal{V}$-continuity space* [24] if $d \leq \Delta_X$ and $d \leq d;d$, or equivalently, if for all $x,y,z \in X$,

$$d(x,x) = \mathsf{0} \qquad \text{and} \qquad d(x,z) \leq d(x,y) \oplus d(y,z).$$

The *dual* of $(X,d)$ is the $\mathcal{V}$-continuity space $(X,d^*)$ where $d^*(x,y) = d(y,x)$. The *symmetrization* of $(X,d)$ is the space $(X,d^s)$ with $d^s(x,y) = d(x,y) \vee d^*(x,y)$. We say that $(X,d)$ is *symmetric* if $d = d^*$.

**Remark 3.2.** Recall that omission of the metric symmetry axiom $d(x,y) = d(y,x)$ is standardly designated by the prefix 'quasi-' and omission of the antisymmetry axiom $d(x,y) = \mathsf{0} \Rightarrow x = y$ by the prefix 'pseudo-'; thus, continuity spaces could be termed *generalized pseudo-quasimetric spaces*, and symmetric continuity spaces *generalized pseudometric spaces.*

The co-quantale $\mathcal{V}$ itself is made into a $\mathcal{V}$-continuity space $(V, d_{\mathcal{V}})$ using the operator $\ominus$:

$$d_{\mathcal{V}}(a,b) = a \ominus b.$$

For any set $A$, the *supremum distance* between $\mathcal{V}$-valued maps $f,g\colon A \to V$ is

$$d_{\mathcal{V}}^{\vee}(f,g) = \bigvee_{a \in A} d_{\mathcal{V}}(f(a), g(a)).$$

The usual notion of nonexpansive map generalizes as expected:

**Definition 3.3 (Nonexpansive maps).** A map $f\colon X \to Y$ between $\mathcal{V}$-continuity spaces $(X, d_1)$ and $(Y, d_2)$ is *nonexpansive* if $d_2(f(x), f(y)) \le d_1(x, y)$ for all $x, y \in X$. We denote the space of nonexpansive maps between $(X, d_1)$ and $(Y, d_2)$ by $(X, d_1) \to_1 (Y, d_2)$. In the special case of nonexpansive $\mathcal{V}$-valued maps we write $\mathsf{Pred}(X, d) = (X, d) \to_1 (V, d_{\mathcal{V}})$.

Ultimately we are interested in defining and reasoning about *behavioural distances.* Generally speaking, a behavioural distance is a $\mathcal{V}$-continuity space defined on the carrier of a $T$-coalgebra $\alpha\colon A \to TA$ in such a way that the behaviour defined by the coalgebra map $\alpha$ is incorporated into the distance values of states in $A$. This is accomplished using *relation liftings*, which lift $\mathcal{V}$-valued relations giving distances between states to those giving distances between successor structures of states. We specifically generalize the notion of nonexpansive lax extension [56] to the quantale-valued case:

**Definition 3.4 (Lax Extension).** A *nonexpansive lax extension* of $T$ is a mapping $L$ that maps $\mathcal{V}$-valued relations $R\colon A \times B \to V$ to relations $LR\colon TA \times TB \to V$ and satisfies the following axioms:

$$
\begin{aligned}
&(L1)\quad R_1 \le R_2 \implies LR_1 \le LR_2\\
&(L2)\quad L(R;S) \le LR;LS\\
&(L3)\quad L\mathrm{Gr}_f \le \mathrm{Gr}_{Tf}\\
&(L4)\quad L\varDelta_{\varepsilon,A} \le \varDelta_{\varepsilon,TA}
\end{aligned}
$$

for all $R, R_1, R_2\colon A \nrightarrow B$, $S\colon B \nrightarrow C$, $f\colon A \to B$ and $\varepsilon \in V$.

(The notion of *lax extension*, given by axioms $(L1)$–$(L3)$, is standard, e.g. [31]; the axiom $(L4)$, introduced in [56], guarantees nonexpansiveness w.r.t. the supremum metric as shown in Lemma 3.6.)

**Lemma 3.5.** *If $L$ is a lax extension of $T$ and $(A, d)$ is a $\mathcal{V}$-continuity space, then so is $(TA, Ld)$.*

**Lemma 3.6.** *If $L$ is a nonexpansive lax extension of $T$, then $L$ is in fact nonexpansive w.r.t. the supremum metric. That is, for $R_1, R_2\colon A \nrightarrow B$ we have $d_{\mathcal{V}}^{\vee}(LR_1, LR_2) \le d_{\mathcal{V}}^{\vee}(R_1, R_2)$.*

*Proof.* We have $d_{\mathcal{V}}^{\vee}(R_1, R_2) \le \varepsilon \iff R_1 \le R_2; \varDelta_\varepsilon$. Using $(L1)$, $(L2)$ and $(L4)$, we have $LR_1 \le L(R_2; \varDelta_\varepsilon) \le LR_2; L\varDelta_\varepsilon \le LR_2; \varDelta_\varepsilon$, so $d_{\mathcal{V}}^{\vee}(LR_1, LR_2) \le \varepsilon$. □

For technical purposes, we will be interested in a generalized version of total boundedness (recall that a standard metric space is compact iff it is complete and totally bounded):

**Definition 3.7 (Total boundedness).** Let $(X, d)$ be a $\mathcal{V}$-continuity space. For $\varepsilon \gg \mathsf{0}$, we write $B_\varepsilon^s(x) = \{y \in X \mid d^s(x, y) \le \varepsilon\}$ for the *(symmetric) ball* of radius $\varepsilon$ around $x \in X$. A *finite $\varepsilon$-cover* of $(X, d)$ is a choice of finitely many $x_1, \dots, x_n \in X$ such that $X = \bigcup_{i=1}^n B_\varepsilon^s(x_i)$. We say that $(X, d)$ is *totally bounded* if $X$ has a finite $\varepsilon$-cover for each $\varepsilon \gg \mathsf{0}$.

**Remark 3.8.** Note that use of the symmetrization $d^s$ is essential in the above definition; e.g. in the unit interval, with $d(x, y) = x \ominus y$, the set $\{y \mid d(0, y) \leq \varepsilon\}$ is the whole space, so 0 alone would form an $\varepsilon$-cover of $[0, 1]$ if we replaced $d^s$ with $d$.

Moreover, our main result involves a generalization of the standard notion of density:

**Definition 3.9 (Density).** Let $(X, d)$ be a $\mathcal{V}$-continuity space. A subset $Y \subseteq X$ is *dense* if for every $x \in X$ and $\varepsilon \gg 0$ there exists $y \in Y$ such that $d^s(x, y) \leq \varepsilon$.

**Assumption 3.10.** Throughout the paper, we *fix a value co-quantale $\mathcal{V}$ that is totally bounded as a $\mathcal{V}$-continuity space.* Moreover, we fix a dense subset $V_0 \subseteq V$ for use as a set of truth constants in the relevant logics, with a view to keeping the syntax countable in the central examples. (The technical development, on the other hand, does not require $V_0$ to be countable, so we can always take $V_0 = V$.)

**Example 3.11 ((Value) co-quantales).**

1. The set $2 = \{0, 1\}$, with $0 \leq 1$ and with binary join as the monoid structure, is a value co-quantale [24], and of course totally bounded. 2-Continuities $d$ are just preorders, with $y$ being above $x$ if $d(x, y) = 0$ (!); symmetric 2-continuities are equivalence relations. Notice that $0 \gg 0$ in 2. The $\ominus$ operator is given by $a \ominus b = 1$ iff $a = 1$ and $b = 0$.
2. The dual of every *locale* (e.g. [8]), in particular the set of closed subsets of any topological space, forms a co-quantale, with binary join as the monoid structure. However, locales are not in general value co-quantales. The dual $\Omega(R)$ of the *free* locale over a set $R$, described as the lattice of downclosed systems of finite subsets of $R$ (ordered by reverse inclusion of such set systems), does form a value co-quantale [24], and is totally bounded [30]. $\Omega(R)$-continuity spaces are known as *structure spaces* [30,24].
3. The unit interval $[0, 1]$ is totally bounded. $[0, 1]$-Continuity spaces coincide with 1-bounded pseudo-quasimetric spaces, and symmetric $[0, 1]$-continuity spaces with 1-bounded pseudometric spaces in the standard sense (cf. Remark 3.2).
4. *Convex-nondeterministic distances:* The set $\mathcal{I}$ of nonempty closed subintervals (i.e. finitely generated nonempty convex subsets) of $[0, 1]$, written in the form $[a, b]$ with $a \leq b$, ordered by $[a, b] \leq [c, d]$ iff $a \leq c$ and $b \leq d$, and equipped with truncated Minkowski addition $[a, b] \oplus [c, d] = [a \oplus c, b \oplus d]$ (with $\oplus$ on $[0, 1]$ defined as in the previous item), is a totally bounded value co-quantale. We write $\llbracket a, b \rrbracket = [a, \max(a, b)]$. We have $[a, b] \gg 0 = [0, 0]$ iff $a > 0$, and $[a, b] \ominus [c, d] = \llbracket a \ominus c, b \ominus d \rrbracket$, again with $\ominus$ on $[0, 1]$ described as in the previous item. We can think of an $\mathcal{I}$-continuity space as assigning to each pair of points a nondeterministic distance, given as an interval of possible distances.

## 4 Quantale-Valued Modal and Predicate Logics

We next introduce the main objects of study, quantale-valued coalgebraic modal and predicate logics. They will feature modalities interpreted using a quantitative version of *predicate liftings* [45,50,51]. Predicate liftings take their name from the fact that they lift predicates on a base set $X$ to predicates on the set $TX$ (where $T$ is our globally fixed functor representing the system type according to Section 2). We work with *$\mathcal{V}$-valued predicates*, which are organized in the *contravariant $\mathcal{V}$-powerset* functor $\mathcal{Q}$ given on sets $X$ by $\mathcal{Q}X = X \to V$ and on functions $f\colon X \to Y$ by $\mathcal{Q}f(g) = g \circ f$ (that is, $\mathcal{Q}$ is a functor $\mathsf{Set}^{\mathrm{op}} \to \mathsf{Set}$ where $\mathsf{Set}^{\mathrm{op}}$ is the opposite category of $\mathsf{Set}$). In keeping with the prevalent reading in fuzzy and probabilistic logics (where, typically, $\mathcal{V} = [0,1]$), we read $\mathsf{0} \in \mathcal{V}$ as 'false' and $\mathsf{1} \in \mathcal{V}$ as 'true' (opposite choices are also found in the literature, e.g. in modal logics for metric transition systems [3], where $0 \in [0,1]$ is interpreted as 'true'). Predicate liftings can have arbitrary finite arities [50]. For brevity, we restrict the presentation to unary modalities and predicate liftings; generalizing to higher arities requires only more indexing.

**Definition 4.1.** A *($\mathcal{V}$-valued) predicate lifting* is a natural transformation $\lambda\colon \mathcal{Q} \to \mathcal{Q} \circ T$, i.e. a family of maps $\lambda_X\colon \mathcal{Q}X \to \mathcal{Q}TX$, indexed over all sets $X$, such that $\lambda_Y(f)(Th(t)) = \lambda_X(f \circ h)(t)$ for all $f\colon Y \to V$, $h\colon X \to Y$, $t \in TX$.

**Definition 4.2.** Let $\lambda$ be a predicate lifting.

1. $\lambda$ is *monotone* if for all sets $X$ and all $f, g \in \mathcal{Q}X$ with $f \le g$ we have $\lambda_X(f) \le \lambda_X(g)$.
2. $\lambda$ is *nonexpansive* if for all sets $X$ and all $f, g \in \mathcal{Q}X$ we have $d_{\mathcal{V}}^{\vee}(\lambda_X(f), \lambda_X(g)) \le d_{\mathcal{V}}^{\vee}(f, g)$.

For the remainder of the paper, we *fix a set $\Lambda$ of monotone and nonexpansive predicate liftings*, which, by abuse of notation, we also use as modalities in the syntax. A basic example is the $\Diamond$ modality of quantitative probabilistic modal logic [10], which denotes expected probability (in the next transition step) and corresponds to a predicate lifting for the (sub-)distribution functor $\mathcal{S}$ (Section 2); see Example 4.4.2 for details. The generic **syntax** of *($\mathcal{V}$-valued) quantitative coalgebraic modal logic* is then given by the grammar

$$\varphi, \psi ::= c \mid \varphi \oplus c \mid \varphi \ominus c \mid \varphi \wedge \psi \mid \varphi \vee \psi \mid \lambda\varphi \qquad (c \in V_0, \lambda \in \Lambda).$$

The operators $\oplus$, $\ominus$, $\vee$, $\wedge$ denote co-quantale operations, the meaning of $\lambda$ is determined by the associated predicate lifting. As usual, the *rank* of a formula $\varphi$ is the maximal nesting depth of modalities $\lambda$ in $\varphi$. We denote the set of all modal formulae by $\mathcal{L}^{\Lambda}$ and the set of formulae of rank at most $n$ by $\mathcal{L}_n^{\Lambda}$.

Formally, the **semantics** is defined by assigning to each formula $\varphi$ and each $T$-coalgebra $\alpha\colon A \to TA$ the *extension* $[\![\varphi]\!]_\alpha\colon A \to V$, or just $[\![\varphi]\!]$, of $\varphi$ over $\alpha$, recursively defined by

$$[\![\varphi \oplus c]\!](a) = [\![\varphi]\!](a) \oplus c \qquad [\![\varphi \ominus c]\!](a) = [\![\varphi]\!](a) \ominus c$$
$$[\![\varphi \wedge \psi]\!](a) = [\![\varphi]\!](a) \wedge [\![\psi]\!](a) \qquad [\![\varphi \vee \psi]\!](a) = [\![\varphi]\!](a) \vee [\![\psi]\!](a)$$
$$[\![c]\!](a) = c \qquad [\![\lambda\varphi]\!](a) = \lambda_A([\![\varphi]\!])(\alpha(a))$$

**Remark 4.3.** Fuzzy logics differ widely in their interpretation of propositional connectives (e.g [41]). In our modal syntax, we necessarily restrict to nonexpansive operations, in order to ensure nonexpansiveness w.r.t. behavioural distance later; this is typical of characteristic logics for behavioural distances (such as quantitative probabilistic modal logic [10]). The logic hence does not include binary $\oplus$ or $\ominus$ (in the above syntax, we insist that one of the arguments is a constant). In terminology usually applied to $\mathcal{V} = [0,1]$, we thus allow *Zadeh* connectives (such as $\vee$, $\wedge$) but not *Łukasiewicz* connectives, so for $\mathcal{V} = [0,1]$, the above version of quantitative coalgebraic modal logic is essentially the Zadeh fragment of Łukasiewicz fuzzy coalgebraic modal logic [51].

The syntax does not include negation $1 \ominus (-)$; if $\mathcal{V}$ satisfies the De Morgan laws (e.g. these hold in $[0,1]$), $\Lambda$ is closed under *duals* $1 \ominus (\lambda(1 \ominus (-)))$, and $V_0$ is closed under negation (i.e. $c \in V_0$ implies $1 \ominus c \in V_0$), then negation can be defined via negation normal forms as usual.

As the ambient predicate logic of the above modal logic, we use *($\mathcal{V}$-valued) quantitative coalgebraic predicate logic*, a quantitative variant of two-valued coalgebraic predicate logic [40]. Its **syntax** is given by

$$\varphi, \psi ::= c \mid x = y \mid \varphi \oplus c \mid \varphi \ominus c \mid \varphi \wedge \psi \mid \varphi \vee \psi \mid \exists x.\varphi \mid \forall x.\varphi \mid x\lambda\lceil y\colon \varphi\rceil$$

where $c \in V_0$, $\lambda \in \Lambda$, and $x, y$ come from a fixed supply $\mathsf{Var}$ of (individual) variables. The reading of $x\lambda\lceil y\colon \varphi\rceil$ is the modalized truth degree (according to $\lambda$) to which the successors $y$ of a state $x$ satisfy $\varphi$; e.g. with $\Diamond$ as above, $x\Diamond\lceil y\colon \varphi\rceil$ is the expected truth value of $\varphi$ at a random successor $y$ of $x$. The **semantics** over $(A, \alpha)$ as above is given by $\mathcal{V}$-valued maps $[\![\varphi]\!]_\alpha$, or just $[\![\varphi]\!]$, that are defined on valuations $\kappa\colon \mathsf{Var} \to A$. The interesting clauses in the definition are

$$[\![\exists x.\varphi]\!](\kappa) = \bigvee_{a \in A} [\![\varphi]\!](\kappa[x \mapsto a]) \qquad [\![\forall x.\varphi]\!](\kappa) = \bigwedge_{a \in A} [\![\varphi]\!](\kappa[x \mapsto a])$$
$$[\![x\lambda\lceil y\colon \varphi\rceil]\!](\kappa) = \lambda_A([\![\varphi]\!](\kappa[y \mapsto \cdot]))(\alpha(\kappa(x)))$$

(where $\kappa[y \mapsto a]$ maps $y$ to $a$ and otherwise behaves like $\kappa$, and by $[\![\varphi]\!](\kappa[y \mapsto \cdot])$ we mean the predicate that maps $a$ to $[\![\varphi]\!](\kappa[y \mapsto a])$). Moreover, equality is crisp, i.e. $[\![x = y]\!](\kappa)$ is $\mathsf{1}$ if $\kappa(x) = \kappa(y)$, and $\mathsf{0}$ otherwise.

**Example 4.4.** We discuss some instances of the above framework.

1. *Fuzzy modal logic:* Take $T$ to be the *covariant* $\mathcal{V}$-valued powerset functor, i.e. $TX = X \to V$ and $Tf(A)(y) = \bigvee\{A(x) \mid f(x) = y\}$ for $f : X \to Y$. We think of $A \in TX$ as a $\mathcal{V}$-valued fuzzy subset of $X$; we say that $A$ is *crisp* if $A(x) \in \{\mathsf{0}, \mathsf{1}\}$ for all $x$. Put $\Lambda = \{\Diamond\}$ where $\Diamond_X(A)(B) = \bigvee\{A(x) \wedge B(x) \mid x \in X\}$ for $A \in \mathcal{Q}X$, $B \in TX$. Then $T$-coalgebras are equivalent to fuzzy Kripke frames, which consist of a set $X$ and a fuzzy relation $R\colon X \times X \to V$, and $\Diamond$ is the natural fuzzification of the standard diamond modality. Fuzzy propositional atoms from a set $\mathsf{At}$

can be added by passing to the functor that maps a set $X$ to $\mathcal{Q}(\mathsf{At}) \times TX$. Instantiating to $\mathcal{V} = [0,1]$, we obtain a basic modal logic of fuzzy relations, or in description logic terminology *Zadeh fuzzy* $\mathcal{ALC}$ [53]. The corresponding instance of quantitative coalgebraic predicate logic is essentially the Zadeh fragment of Novak's Łukasiewicz fuzzy first order logic [43].

2. *Probabilistic modal logic:* As indicated in Section 2, coalgebras for the subdistribution functor $\mathcal{S}$ are probabilistic transition systems (with possible deadlock). We take $\mathcal{V} = [0,1]$ and $\Lambda = \{\Diamond\}$, interpreted by the predicate lifting

$$\Diamond_X(A)(\mu) = \mathbb{E}_\mu(A) \qquad \text{for } \mu \in \mathcal{S}X$$

where $\mathbb{E}_\mu(A)$ denotes the expected value of $A(x)$ when $x$ is distributed according to $\mu$. The induced instance of quantitative coalgebraic modal logic is *(quantitative) probabilistic modal logic* [10], which may be seen as a quantitative variant of two-valued probabilistic modal logic [39], and embeds into the probabilistic $\mu$-calculus [34,42]. Propositional atoms are treated analogously as in the previous item (and indeed probabilistic modal logic is trivial without them). The ambient quantitative probabilistic first-order logic arising as the corresponding instance of quantitative coalgebraic predicate logic is a quantitative variant of Halpern's type-1 (i.e. statistical) probabilistic first-order logic [27].

3. *Metric modal logic:* In their simplest form, *metric transition systems* [3] are just transition systems in which states are labelled in a metric space $S$ (numerous variants exist, e.g. with states themselves forming a metric space or with transitions labelled in a metric space [9]). We work with a generalized version where $(S, d_S)$ is a $\mathcal{V}$-continuity space. Metric transition systems are then coalgebras for the functor $TX$ given on sets by $TX = S \times \mathcal{P}X$. We take $\Lambda = \{\Diamond\} \cup S$. We interpret $\Lambda$ using predicate liftings

$$\Diamond_X(A)(s,B) = \bigvee\{A(x) \mid x \in B\} \qquad r_X(A)(s,B) = d_S(s,r)$$

for $A \in \mathcal{Q}X$, $(s,B) \in TX$, $r \in S$. Note that $r \in S$ ignores its argument $A$, so is effectively a nullary modality. Note also that as per our interpretation of truth values, this nullary modality is read as distinctness from $r$; in case $\mathcal{V} = [0,1]$, the degree of equality to $r$ can be expressed as $1 \ominus r$. The induced instance of coalgebraic modal logic is related to characteristic logics for branching-time behavioural distances on metric transition systems [3,9].

4. *Convex-nondeterministic metric modal logic:* We continue to consider metric transition systems as recalled in the previous item, reusing the designators $T, S, d_S$, and taking $\mathcal{V} = [0,1]$ for simplicity. Recall the value co-quantale $\mathcal{I}$ of nonempty closed subintervals of $[0,1]$ from Example 3.11.4. We turn the predicate liftings for $r \in S$ defined in the previous item into $\mathcal{I}$-valued predicate liftings by prolonging them along the inclusion $\iota\colon [0,1] \hookrightarrow \mathcal{I}$, given by $\iota(a) = [a,a]$. We define an $\mathcal{I}$-valued predicate lifting $M$ for $T$, where $\mathcal{I}$ is the value quantale of closed intervals introduced in Example 3.11.4, by

$$M_X(A)(s,B) = [\bigwedge\{\pi_1(A(x)) \mid x \in B\}, \bigvee\{\pi_2(A(x)) \mid x \in B\}]$$

where $\pi_i\colon \mathcal{I} \to [0,1]$ denote the evident *projections* $\pi_1([a,b]) = a$, $\pi_2([a,b]) = b$. That is, $M$ returns the range of truth values that $A$ takes on $B$.

### 5 Behavioural Distance and Quantitative Bisimulation Invariance

The behavioural distance between states of a coalgebra $\alpha\colon A \to TA$ is defined as a least fixpoint that arises from an iterative process: Initially, at depth 0, all states are thought of as equivalent and their distance is therefore $\mathsf{0}$. In order to increase the depth of the behavioural distance from $n$ to $n+1$, we lift the depth-$n$ distance on $A$ to a the set $TA$ of successor structures. Formally, this is accomplished using the following quantale-valued version of the coalgebraic Kantorovich lifting [4,56]:

**Definition 5.1 (Kantorovich lifting).** Let $A$ and $B$ be sets and $R\colon A \nrightarrow B$.

1. A pair $(f, g)$ of functions $f\colon A \to V$, $g\colon B \to V$ is *$R$-nonexpansive* if $f(a) \ominus g(b) \le R(a, b)$ for all $a \in A$, $b \in B$.
2. The *Kantorovich lifting* of $R$ is the relation $K_\Lambda(R)\colon TA \nrightarrow TB$ given by

$$K_\Lambda(R)(t_1, t_2) = \bigvee\{\lambda_A(f)(t_1) \ominus \lambda_B(g)(t_2) \mid \lambda \in \Lambda, (f, g)\ R\text{-nonexpansive}\}.$$

(Here, $\Lambda$ is the set of modalities fixed in Section 4.) Generalizing [56, Theorem 5.6], we have:

**Lemma 5.2.** *The Kantorovich lifting is a nonexpansive lax extension.*

**Example 5.3 (Kantorovich liftings).**

1. For $\mathcal{V} = [0, 1]$ and $\mathcal{V}$-valued *fuzzy modal logic* with $\Lambda = \{\Diamond\}$ (i.e. for simplicity without propositional atoms; cf. Example 4.4.1), the Kantorovich lifting $K_\Lambda(R)$ of a $\mathcal{V}$-valued relation $R\colon X \nrightarrow Y$ coincides with an asymmetric generalized Hausdorff lifting; i.e.

$$K_\Lambda(R)(A, B) = \bigvee_{x\in X} \bigwedge_{y\in Y} ((A(x) \ominus B(y)) \vee (A(x) \wedge R(x, y)))$$

for $A \in TX = X \to V$, $B \in TY$. (Obtaining a similar description for general $\mathcal{V}$ remains an open problem.) In particular, on crisp sets $A, B$, the symmetrization $K_\Lambda(R)^s$ is the usual Hausdorff lifting $K_\Lambda(R)^s(A, B) = \max(\bigvee_{A(x)=1} \bigwedge_{B(y)=1} R(x, y), \bigvee_{B(y)=1} \bigwedge_{A(x)=1} R(x, y))$.

2. For *probabilistic modal logic* (Example 4.4.2), the restriction of $K_\Lambda$ to distributions coincides, by definition, with the usual (symmetric) Kantorovich-Wasserstein lifting (e.g. [10]). On subdistributions, one obtains an asymmetric variant, whose symmetrization then coincides with the standard one.

3. For $\mathcal{V}$-valued *metric modal logic* (Example 4.4.3), with $\Lambda = \{\Diamond\} \cup S$, we similarly obtain a $\mathcal{V}$-valued (asymmetric) Hausdorff distance

$$K_\Lambda(R)((s, A), (t, B)) = d(s, t) \vee \bigvee_{x\in A} \bigwedge_{y\in B} R(x, y)$$

on $(s, A) \in TX = S \times \mathcal{P}(X)$, $(t, B) \in TY$, and $R\colon X \nrightarrow Y$; a characterization that in this case holds for unrestricted $\mathcal{V}$.

4. *Convex-nondeterministic metric modal logic:* The $\mathcal{I}$-valued Kantorovich lifting induced by the set $\Lambda = \{M\} \cup S$ of modalities on metric transition systems, with notation as in Examples 3.11.4 and 4.4.4, is given by

$$K_\Lambda(R)((s,A),(t,B)) = \iota(d(s,t)) \vee \llbracket \bigvee_{y \in B} \bigwedge_{x \in A} \pi_1(R(x,y)), \bigvee_{x \in A} \bigwedge_{y \in B} \pi_2(R(x,y)) \rrbracket$$

on $(s,A) \in TX = S \times \mathcal{P}(X)$, $(t,B) \in TY$, and $R \colon X \nrightarrow Y$ (recall that the $\pi_i$ are the projections $\mathcal{I} \to [0,1]$, and $\iota \colon [0,1] \to \mathcal{I}$ denotes the evident injection).

For purposes of lifting $\mathcal{V}$-continuity structures as relations, nonexpansive pairs can be replaced with the more familiar notion of nonexpansive map:

**Lemma 5.4.** *Let $(A,d)$ be a $\mathcal{V}$-continuity space and let $(f,g)$ be $d$-nonexpansive. Put $h(b) = \bigvee_{a \in A} f(a) \ominus d(a,b)$. Then $f \leq h \leq g$ and $h \in \mathsf{Pred}(A,d)$.*

By monotonicity of predicate liftings we get the following alternative formulation for the Kantorovich lifting of a $\mathcal{V}$-continuity structure:

**Lemma 5.5.** *Let $(A,d)$ be $\mathcal{V}$-continuity space. Then for all $t_1, t_2 \in TA$*

$$K_\Lambda(d)(t_1,t_2) = \bigvee \{\lambda_A(h)(t_1) \ominus \lambda_A(h)(t_2) \mid \lambda \in \Lambda, h \in \mathsf{Pred}(A,d)\}.$$

Using the Kantorovich lifting, we can now define a sequence of behavioural distances between states $a,b$ in a $T$-coalgebras $\alpha \colon A \to TA$, $\beta \colon B \to TB$:

$$d_0^K(a,b) = \mathsf{0} \quad d_{n+1}^K(a,b) = K_\Lambda(d_n^K)(\alpha(a),\beta(b)) \quad d_\omega^K(a,b) = \bigvee_{n<\omega} d_n^K(a,b).$$

By general fixed point theory, the continuation of this ordinal-indexed sequence past $\omega$ eventually stabilizes, that is, there exists some ordinal $\gamma$ such that $d_{\gamma+1}^K = d_\gamma^K$. The arising least fixed point is the unbounded *behavioural distance* $d^K$, alternatively given by

$$d^K = \bigwedge \{d \mid d = K_\Lambda(d) \circ (\alpha \times \beta)\}.$$

These behavioural distances lead to an appropriate generalization of the notion of bisimulation invariance. A family $f$ of $\mathcal{V}$-valued predicates $f_\alpha$ indexed over $T$-coalgebras $\alpha \colon A \to TA$ – such as the extension of a modal formula or of a first-order formula with a single free variable – is said to be *behaviourally nonexpansive* if it is nonexpansive with respect to behavioural distance $d^K$, i.e. if for all coalgebras $\alpha \colon A \to TA$, $\beta \colon B \to TB$ and all $a \in A, b \in B$,

$$f_\alpha(a) \ominus f_\beta(b) \leq d^K(a,b). \tag{1}$$

Similarly, $f$ is *depth-$n$ behaviourally nonexpansive* for finite depth $n$ if $f$ is nonexpansive with respect to depth-$n$ behavioural distance $d_n^K$.

To match these notions to the classical setting, consider the binary coquantale 2. In the general case, the above notion of behavioural nonexpansiveness should then be thought of as preservation under simulation: States $a,b$ have (asymmetric) distance $\mathsf{0}$ if $b$ simulates $a$, and in this case, (1) stipulates that if $f$ is true at $a$, then $f$ is also true at $b$.

**Example 5.6.** The behavioural distance arising from the Kantorovich lifting of metric modal logic (Example 5.3.3) is a *simulation distance*. The value $d^K(a, b)$ quantifies the degree to which traces starting at $b$ simulate traces starting at $a$, where the distance from one trace to another is the supremum over the distances at all time steps.

On the other hand, there are many cases where the behavioural distance $d^K$ is symmetric. If $\mathcal{V} = [0, 1]$ and the set $\Lambda$ is closed under duals (Remark 4.3), then we have that $K_\Lambda(R^*) = K_\Lambda(R)^*$ for all $R$ and therefore $d^K$ is symmetric [56]. Concretely, if we put $\Box_X(A) = 1 \ominus \Diamond_X(1 \ominus A)$, then in the case of fuzzy modal logic (Example 4.4.1) we have $\Box_X(A)(B) = \bigwedge\{(1 \ominus B(x)) \vee A(x) \mid x \in X\}$ and in the case of probabilistic modal logic (Example 4.4.2) we have $\Box_X(A)(\mu) = \mathbb{E}_\mu(A) \oplus (1 \ominus \mu(X))$, and in both cases $\Lambda = \{\Diamond, \Box\}$ yields a symmetric distance.

In these symmetric cases distance $\mathsf{0}$ determines a notion of *bi*similarity, and behavioural nonexpansiveness amounts to the standard notion of bisimulation invariance. Thus, the following straightforward lemma generalizes both bisimulation invariance of modal logic and preservation of positive modal logic (with only diamond modalities) under simulation:

**Lemma 5.7.** *All modal formulae are behaviourally nonexpansive, and all modal formulae of rank at most $n$ are depth-$n$ behaviourally nonexpansive.*

As expected, coalgebra morphisms preserve behaviour on the nose:

**Lemma 5.8.** *Let $\alpha\colon A \to TA$ and $\beta\colon B \to TB$ be coalgebras and $h\colon A \to B$ a coalgebra morphism, that is $Th \circ \alpha = \beta \circ h$. Then $d^{K,s}(a, h(a)) = \mathsf{0}$ for all $a \in A$.*

Another way to define distances between states of a coalgebra is in terms of the modal formulae:

**Definition 5.9 (Logical distance).** Let $a, b$ be states in coalgebras $\alpha\colon A \to TA, \beta\colon B \to TB$. We define

$$d^L_n(a, b) = \bigvee\{[\![\varphi]\!](a) \ominus [\![\varphi]\!](b) \mid \varphi \in \mathcal{L}^\Lambda_n\}$$

$$d^L(a, b) = \bigvee\{[\![\varphi]\!](a) \ominus [\![\varphi]\!](b) \mid \varphi \in \mathcal{L}^\Lambda\}$$

The relationship between fixpoint-based distances $d^K$ and logical distances $d^L$ is at the heart of the study of behavioural nonexpansiveness and modal expressiveness. For instance, Lemma 5.7 can equivalently be expressed by the inequalities $d^L \leq d^K$ and $d^L_n \leq d^K_n, n < \omega$. In Section 6, we investigate the converse inequalities.

### 6 Modal Approximation

We now establish our first contribution, a quantitative coalgebraic Hennessy-Milner theorem. To this end, we first need to pin down the exact relationship of the two families of distances at finite depth.

**Theorem 6.1.** *Let the set $\Lambda$ of monotone and nonexpansive predicate liftings from Section 4 be finite and let $(A, \alpha)$ be a coalgebra. For all $n < \omega$:*

1. *We have $d_n^K = d_n^L =: d_n$*
2. *The space $(A, d_n)$ is totally bounded.*
3. *The set $\mathcal{L}_n^\Lambda$ is a dense subset of* $\mathsf{Pred}(A, d)$.

**Remark 6.2.** The need for assuming that the set $\Lambda$ of modalities is finite is specific to quantitative Hennessy-Milner theorems (and implicitly present also in the existing $[0, 1]$-valued version of the theorem [37]), and not needed in the two-valued case [45,50]. It relates to the total boundedness claim in Theorem 6.1, and features also in the van Benthem theorem, where in fact it is needed also in the two-valued case [52]; indeed, proofs of the original van Benthem theorem start by assuming, in that case w.l.o.g., that there are only finitely many propositional atoms and relational modalities. In our running examples, only the ones featuring metric transition systems are affected by this assumption; indeed, for our theorems to apply to such systems, the space of labels needs to be finite.

Theorem 6.1 is proven by induction on $n$ and most of Section 6 is devoted to the inductive step (the base case $n = 0$ is immediate from $d_0^K = d_0^L = \mathsf{0}$). We fix a coalgebra $\alpha\colon A \to TA$ and an integer $n > 1$ and assume as the inductive hypothesis that the three items of Theorem 6.1 have already been proven for all $m < n$. We show Item 1 in Lemma 6.3, Item 2 in Lemma 6.6, and Item 3 in Lemma 6.7.

**Lemma 6.3.** *We have $d_n^K = d_n^L$ on $A$.*

*Proof (sketch).* We use the alternative formula for the Kantorovich lifting as given in Lemma 5.5. By Item 3 of the inductive hypothesis, and because the predicate liftings are nonexpansive, the maps $\lambda(f) \circ \alpha$ with $f \in \mathsf{Pred}(A, d_{n-1})$ can be approximated using formula expansions $[\![\lambda\psi]\!]$ with $\psi \in \mathcal{L}_{n-1}^\Lambda$. □

Having shown that $d_n^K = d_n^L$, from now on we simply use $d_n$ to denote both. To show that $d_n$ is totally bounded, we make use of the following version of the Arzelà-Ascoli theorem [23, Theorem 4.13].

**Lemma 6.4 (Arzelà-Ascoli).** *Let $(X, d_1)$ and $(Y, d_2)$ be totally bounded $\mathcal{V}$-continuity spaces. Then the space $(X, d_1) \to_1 (Y, d_2)$ is also totally bounded.*

Using Lemma 6.4, we show that the Kantorovich lifting preserves total boundedness; this generalizes a previous result for the case $\mathcal{V} = [0, 1]$ [37, Proposition 29], which in turn generalizes [57, Lemma 5.6].

**Lemma 6.5.** *If the set $\Lambda$ of predicate liftings is finite and $(X, d)$ is a totally bounded $\mathcal{V}$-continuity space, then $(TX, K_\Lambda(d))$ is totally bounded.*

The following is now an easy consequence:

**Lemma 6.6.** *The space $(A, d_n)$ is totally bounded.*

Finally, we show that the modal formulae up to depth $n$ form a dense subspace of the space of all nonexpansive properties:

**Lemma 6.7.** *Let $f \in \mathsf{Pred}(A, d_n)$ be a nonexpansive map and let $\varepsilon \gg 0$. Then there exists some modal formula $\varphi \in \mathcal{L}_n^\Lambda$ such that $d_{\mathcal{V}}^{\vee,s}(f, [\![\varphi]\!]) \leq \varepsilon$.*

*Proof (sketch).* We use the fact that for all $x, y \in A$

$$f(x) = \bigwedge_{y \in A} d_n(x, y) \oplus f(y) = \bigwedge_{y \in A} (\bigvee_{\gamma \in \mathcal{L}_n^\Lambda} [\![\gamma]\!](x) \ominus [\![\gamma]\!](y)) \oplus f(y).$$

The latter term can be approximated using formulae of $\mathcal{L}_n^\Lambda$, where the infimum over $y$ and the supremum over $\gamma$ are made finite using $\varepsilon$-covers of $A$ and $\mathcal{L}_n^\Lambda$. □

Having shown that behavioural distance and logical distance coincide at all finite depths, we are now equipped to prove our first main result, a version of the Hennessy-Milner theorem stating that behavioural distance and logical distance coincide not only at finite depths (Theorem 6.1.1), but in fact also at unbounded depth. In general, this equivalence of distances can only be expected to hold if the functor $T$ in question is *finitary*, or admits approximation by a finitary subfunctor [56]. The functor $T$ is finitary if for all sets $X$ and all $t \in TX$ there exists a finite subset $Y \subseteq X$ such that $t = Ti(s)$ for some $s \in TY$, where $i \colon Y \to X$ is set inclusion. Examples of finitary functors include the *finite powerset functor* $\mathcal{P}_\omega X = \{Y \subseteq X \mid Y \text{ finite}\}$ and the *finite subdistribution functor* $\mathcal{S}_\omega$ which maps a set $X$ to the set of finitely supported probability subdistributions on $X$. König and Mika-Michalski [37] prove a quantitative coalgebraic Hennessy-Milner theorem for the case of the co-quantale $[0, 1]$. We generalize their result as follows:

**Definition 6.8.** We say that the value co-quantale $\mathcal{V}$ is *continuous from below* if for every monotone increasing sequence $(a_n)_{n<\omega}$ in $\mathcal{V}$ and every $\varepsilon \gg 0$, there exists some $n$ such that $a_n \oplus \varepsilon \geq \bigvee_{n<\omega} a_n$.

This condition essentially allows the use of epsilontic arguments also for joins of increasing sequences, while value co-quantales in general allow this only for meets. It holds in all our running examples.

**Theorem 6.9 (Quantified Hennessy-Milner theorem).** *Let $\Lambda$ be a finite set of monotone and nonexpansive predicate liftings, let $T$ be a finitary functor and let $\mathcal{V}$ be a totally bounded value co-quantale that is continuous from below. Then we have $d^K = d^L$.*

*Proof (sketch).* Because $\mathcal{V}$ is continuous from below, we have $K_\Lambda(d_\omega^K) = \bigvee_{n<\omega} K_\Lambda(d_n^K)$ on finite sets, and as $T$ is finitary, this also holds for all sets. This implies that $d_\omega^K = d_{\omega+1}^K = d^K$, so that

$$d^K = \bigvee_{n<\omega} d_n^K = \bigvee_{n<\omega} d_n^L = d^L. \qquad \square$$

Besides examples already covered by the $[0, 1]$-valued version of the theorem [37], this result instantiates, e.g., to a quantitative Hennessy-Milner theorem for convex-nondeterministic metric modal logic (Example 4.4.4).

### 7 Locality and Modal Characterization

We proceed to establish our main result, the quantitative coalgebraic van Benthem theorem. The main tool in the proof of this result is a notion of *locality*, which characterizes formulae that only depend on the structure of the model in some neighbourhood of the state under consideration. This poses a challenge when it comes to coalgebraic models, as these need not come with a built-in graph structure that could be used to define what it means for two states to be neighbouring. To solve this, we make use of a technique based on *supported* coalgebras that has previously been used in the proof of a two-valued coalgebraic van Benthem theorem [52].

Recall from Section 2 that we assume $T\emptyset \neq \emptyset$. We fix an element $\bot \in T\emptyset$, and for each set $A$ put $\bot_A = Ti(\bot)$, where $i\colon \emptyset \to A$ is the empty map.

**Definition 7.1 (Support).** Let $A$ be a set. We say that a set $B \subseteq A$ is a *support* of $t \in TA$ if $t \in TB$. A *supported coalgebra* is a coalgebra $\alpha\colon A \to TA$ together with a map $\mathrm{supp}_\alpha\colon A \to \mathcal{P}A$ such that $\mathrm{supp}_\alpha(a)$ is a support of $\alpha(a)$ for every $a \in A$.

Every coalgebra can be supported because we can always put $\mathrm{supp}_\alpha(a) = A$ for all $a \in A$. Supporting a coalgebra equips it with a graph structure:

**Definition 7.2 (Neighbourhood).** Let $\mathcal{A} = (A, \alpha, \mathrm{supp}_\alpha)$ be a supported coalgebra.

1. The *Gaifman graph* of $\mathcal{A}$ is the undirected graph with vertex set $A$ and edge set $\{\{a, b\} \mid b \in \mathrm{supp}_\alpha(a)\}$.
2. For any $a, b \in A$, the *Gaifman distance* $D_{\mathrm{supp}}(a, b)$ is the least number of steps to get from $a$ to $b$ in the Gaifman graph (or $\infty$, if no path from $a$ to $b$ exists).
3. The *radius-k neighbourhood* of a state $a \in A$ is the set $U^k(a) = \{b \in A \mid D_{\mathrm{supp}}(a, b) \leq k\}$.

For any $k < \omega$ and any state $a$ in a supported coalgebra $\mathcal{A} = (A, \alpha, \mathrm{supp}_\alpha)$, we can define a supported coalgebra $\mathcal{A}_a^k = (U^k(a), \alpha^k, \mathrm{supp}_{\alpha^k})$ on the radius-$k$ neighbourhood of $a$. The coalgebra map $\alpha^k\colon U^k(a) \to T(U^k(a))$ is given by $\alpha^k(b) = \alpha(b)$ if $\mathrm{supp}_\alpha(b) \subseteq U^k(a)$ and $\alpha^k(b) = \bot_A$ otherwise. We note that the latter case only occurs for states on the edge of $U^k(a)$, that is when $D_{\mathrm{supp}}(a, b) = k$. Note that $\bot_A$ has empty support by construction, so that we can put $\mathrm{supp}_{\alpha^k}(b) = \emptyset$ in this latter case and $\mathrm{supp}_{\alpha^k}(b) = \mathrm{supp}_\alpha(b)$ otherwise.

Using the neighbourhood around a state and the coalgebra structure defined on it, we can now define our notion of locality:

**Definition 7.3.** A formula $\varphi$ is *k-local* if we have $[\![\varphi]\!]_\alpha(a) = [\![\varphi]\!]_{\alpha^k}(a)$ for all supported coalgebras $\mathcal{A} = (A, \alpha, \mathrm{supp}_\alpha)$ and all $a \in A$.

**Lemma 7.4.** *For every supported coalgebra $\mathcal{A} = (A, \alpha, \mathrm{supp}_\alpha)$, $k < \omega$ and $a \in A$, we have $d_k^{K,s}(a, a) = 0$, where the first $a$ lives in $\mathcal{A}$ and the second in $\mathcal{A}_a^k$.*

A key step in the proof is the following locality result, which in similar form appears also in proofs of the classical van Benthem theorem [44], and is proved, in our case, by a game-theoretic method that is related to classical Ehrenfeucht-Fraïssé games:

**Lemma 7.5.** *Let $\varphi(x)$ be a behaviourally nonexpansive formula with $\mathsf{qr}(\varphi) \leq n$. Then $\varphi$ is $k$-local for $k = 3^n$.*

*Proof (sketch).* Consider a spoiler-duplicator game over $n$ rounds, where both players place a pebble every round and the second player needs to maintain the invariant that if there are $m$ rounds remaining the radius $3^m$ neighbourhoods around the pebbles need to be isomorphic. One can show that this invariant guarantees equivalence on formulae of rank at most $m$.

We use this game to prove for every supported coalgebra $\mathcal{A}$ that $\varphi$ has the same value on $\mathcal{A}$ and $\mathcal{A}_a^k$. Nonexpansiveness of $\varphi$ is used to extend the two coalgebras in such a way that the duplicator always has a suitable response. □

We next show that every nonexpansive formula that is local is also nonexpansive at some finite depth. We make use of an unravelling construction, where a coalgebra is enlarged so that the successors of every state in the unravelling (as given by the support relation) form a tree.

**Definition 7.6 (Unravelling).** The *unravelling* of a supported coalgebra $\mathcal{A} = (A, \alpha, \mathrm{supp}_\alpha)$ is the supported coalgebra $\mathcal{A}^* = (A^+, \alpha^*, \mathrm{supp}_{\alpha^*})$, where $A^+$ is the set of nonempty sequences over $A$ and for $a_1 \dots a_n \in A^+$ we have $\alpha^\star(a_1 \dots a_n) = Tf(\alpha(a_n))$ and $\mathrm{supp}_{\alpha^*}(a_1 \dots a_n) = f[\mathrm{supp}_\alpha(a_n)]$, where $f\colon A \to A^+, a \mapsto a_1 \dots a_n a$.

**Lemma 7.7.** *For every supported coalgebra $\mathcal{A} = (A, \alpha, \mathrm{supp}_\alpha)$ and every $a \in A$, we have $d^{K,s}(a, a) = 0$, where the first $a$ lives in $\mathcal{A}$ and the second in $\mathcal{A}^*$.*

The mentioned nonexpansiveness at finite depth follows:

**Lemma 7.8.** *Let $\varphi$ be behaviourally nonexpansive and $k$-local. Then $\varphi$ is also depth-$k$ behaviourally nonexpansive.*

*Proof (sketch).* By the assumptions on $\varphi$ we may pass from any supported coalgebra to the radius-$k$ neighbourhood in the unravelling, which is shaped like a tree of depth $k$. Between any two such tree structures we have $d_k^K = d^K$, as their behaviour past depth $k$ is fully characterized by the default value $\bot \in T\emptyset$. □

The target result then follows by combining the above lemmas with Theorem 6.1 and a final chain argument that allows us to detach the technical development from the choice of a fixed coalgebra:

**Theorem 7.9 (Quantified van Benthem theorem).** *Let $\Lambda$ be a finite set of monotone and nonexpansive predicate liftings, let $T$ be a standard functor with $T\emptyset \neq \emptyset$, and let $\mathcal{V}$ be a totally bounded value co-quantale. Then for every behaviourally nonexpansive formula $\varphi$ of quantitative coalgebraic predicate logic with quantifier rank at most $n$ and every $\varepsilon \gg 0$ there exists a modal formula $\psi \in \mathcal{L}^\Lambda$ such that for all coalgebras $\alpha\colon A \to TA$ and all $a \in A$, $d_{\mathcal{V}}^s(\llbracket\varphi\rrbracket_\alpha(a), \llbracket\psi\rrbracket_\alpha(a)) \leq \varepsilon$ and the modal rank of $\psi$ is bounded by $3^n$.*

*Proof (sketch).* Using the final chain $(T^n 1)_{n<\omega}$, where 1 is a singleton set, we can construct a coalgebra $(Z, \zeta)$ such that for all $(A, \alpha)$ and all $\varphi, \psi$ we have $d_{\mathcal{V}}^{\vee,s}([\![\varphi]\!]_\alpha, [\![\psi]\!]_\alpha) \leq d_{\mathcal{V}}^{\vee,s}([\![\varphi]\!]_\zeta, [\![\psi]\!]_\zeta)$.

As $\varphi$ is behaviourally nonexpansive, we get that it is also depth-$k$ behaviourally nonexpansive for $k = 3^{\mathsf{qr}(\varphi)}$ by Lemmas 7.5 and 7.8, and by Theorem 6.1.3 for every $\varepsilon \gg 0$ there is $\psi \in \mathcal{L}_k^\Lambda$ such that $d_{\mathcal{V}}^{\vee,s}([\![\varphi]\!]_\zeta, [\![\psi]\!]_\zeta) \leq \varepsilon$. □

To our best knowledge, the only previously known instances of this result in the real-valued setting are the ones for $[0, 1]$-valued fuzzy modal logic [57] and for quantitative probabilistic modal logic [58]. In the two-valued setting, we cover a previous coalgebraic van Benthem result [52] by instantiating to $\mathcal{V} = 2$, and in fact obtain an additional asymmetric version, characterizing fragments that are preserved under simulation. In our running examples, we obtain new concrete van Benthem theorems for $[0, 1]$-valued metric modal logic (Example 4.4.3) and convex-nondeterministic metric modal logic (Example 4.4.4). We cover, by default, the asymmetric case (to be thought of as characterizing fragments that are preserved under quantitative simulation) and, in the cases $\mathcal{V} = [0, 1]$ and $\mathcal{V} = 2$, also the symmetric case (to be thought of as characterizing fragments that are invariant under bisimulation).

## 8 Conclusions

We have established a highly general quantitative version of van Benthem's modal characterization theorem, stating that given a value quantale $\mathcal{V}$ that is totally bounded and continuous from below, all state properties, in a given type of quantitative systems, that are nonexpansive w.r.t. $\mathcal{V}$-valued behavioural distance and expressible in $\mathcal{V}$-valued coalgebraic (first-order) predicate logic can be approximated by $\mathcal{V}$-valued modal formulae of bounded rank. A key technical tool in the proof are versions of the classical Arzela-Ascoli and Stone-Weierstraß theorems for totally bounded quantale-valued (pseudo-quasi-)metric spaces. Coalgebraic generality implies that this result not only subsumes existing quantitative van-Benthem type theorems for fuzzy [57] and probabilistic [58] systems, but we also obtain new results, e.g. for metric transition systems. Via the additional parametrization over a value quantale, we moreover obtain, e.g., a van Benthem theorem for convex-nondeterministic behavioural distance ('states $x, y$ have distance between $a$ and $b$') on metric transition systems. Our result complements previous coalgebraic results for two-valued logics [52]. We do leave some open problems, in particular to determine whether the main result can be sharpened to exact modal expressibility instead of approximability, and to obtain a quantitative modal characterization over finite models, in generalization of Rosen's finite-model variant of van Benthem's theorem [48].

**Acknowledgements** We wish to thank Barbara König for valuable discussions.
